# Impact of Alcohol Policies on Suicidal Behavior: A Systematic Literature Review

**DOI:** 10.3390/ijerph17197030

**Published:** 2020-09-25

**Authors:** Kairi Kõlves, Kate M. Chitty, Rachmania Wardhani, Airi Värnik, Diego de Leo, Katrina Witt

**Affiliations:** 1 Australian Institute for Suicide Research and Prevention, WHO Collaborating Centre for Research and Training in Suicide Prevention, School of Applied Psychology, Griffith University, Brisbane, QLD 4122, Australia; r.wardhani@griffith.edu.au (R.W.); d.deleo@griffith.edu.au (D.d.L.); 2 Clinical Pharmacology and Toxicology Research Group, Discipline of Pharmacology, Faculty of Medicine and Health, University of Sydney, Sydney, NSW 2008, Australia; kate.chitty@sydney.edu.au; 3 School of Natural Sciences and Health, Tallinn University, 10120 Tallinn, Estonia; varnik.airi@gmail.com; 4 Estonian-Swedish Mental Health and Suicidology Institute, 11615 Tallinn, Estonia; 5 Orygen, Melbourne, VIC 3052, Australia; katrina.witt@orygen.org.au; 6 Centre for Youth Mental Health, University of Melbourne, Melbourne, VIC 3052, Australia

**Keywords:** alcohol policy, suicidal behavior, suicide prevention, systematic literature review

## Abstract

Alcohol consumption has been found to be related to suicidal behavior at the individual and population level, but there is lack of literature reviews on the effect of alcohol policies on suicidal behavior. Therefore, the aim of the current study is to conduct a systematic literature review of the impact of alcohol policies at the population level on suicidal behavior and ideation. We searched the Cochrane CENTRAL, Cochrane DARE, EMBASE, Medline, ProQuest, PsycINFO, PubMed, SCOPUS, and Web of Science electronic databases in March 2019. Papers analyzing alcohol policies limiting alcohol use and studying suicidal behaviors as an outcome measure were included; we identified 19 papers. Although the methods and effect sizes varied substantially in the studies, reducing alcohol often led to reduction in suicidal behavior. Ecological-level studies predominantly investigated the effect of restrictions on alcohol availability and increased cost of alcohol, and the majority presented a reduction in suicides across Western and Eastern Europe, as well as the US. The majority of studies were rated as unclear risk of bias for a number of domains due to a lack of clear reporting. Policies targeting harmful alcohol consumption may contribute towards a reduction in suicidal behavior at the population level.

## 1. Introduction

Alcohol use, both abuse and acute consumption, has been found to be an important contributing factor for suicidal behavior at the individual level [1,2,3,4]. A number of potential mechanisms have been proposed, including increases in impulsivity, aggression, depressive and suicidal thoughts, and feelings of hopelessness, especially if people are predisposed or have depression. In addition to the link on individual level, there is a relationship between alcohol consumption at the population (ecological) level and national suicide rates [1]. It has been suggested that the aggregated-level link between alcohol consumption and suicide depends on a drinking culture; the relationship is strong in predominantly spirit-consuming countries with binge-drinking and intoxication-oriented drinking patterns, rather than in wine-cultures such as Southern Europe [5,6], as has been further evidenced in a recent systematic literature review [1]. Overall, spirits consumption have been linked to higher levels of aggression, emotional responses, and confusion than other alcoholic beverages; therefore, potentially strengthening the relationship between acute alcohol intoxication with distress and negative emotions through constricted thinking and impulsivity to suicide [7].

Considering individual and aggregate level links between alcohol and suicidal behaviors, it would be logical to expect that alcohol policies limiting alcohol use in the population should have the potential to prevent also suicidal behavior [8]. However, there is limited discourse about the topic in suicide prevention. For example, Witt and Lubman [9] highlighted inadequate attention of alcohol and other drug use in Australian suicide prevention strategies. There is also a lack of systematic reviews on the impact of alcohol restrictions on suicidal behavior at the individual and aggregated levels. It has been also noted that participants with alcohol or substance abuse are not included in intervention studies or systematic reviews [9]. Nevertheless, we identified a critical review of alcohol policies, which was limited to suicide only within a restricted timeframe (i.e., 1999–2014) and did not follow PRISMA guidelines or estimate risk of bias [10]. Therefore, we aim to fill the gap and conduct a systematic literature review of the impact of alcohol policies at the population level on suicidal behavior by also addressing the limitations of the earlier critical review.

## 2. Materials and Methods

This systematic review followed the guidance contained in the Preferred Reporting Items for Systematic Reviews and Meta-Analyses (PRISMA) statement [11].

### 2.1. Search Strategy

A comprehensive search of the Cochrane CENTRAL, Cochrane DARE, EMBASE, Medline, ProQuest, PsycINFO, PubMed, SCOPUS, and Web of Science electronic databases was conducted for English-language papers without date restriction until 19 March 2019 according to the protocol (Table 1).

### 2.2. Inclusion and Exclusion Criteria

Inclusion criteria required studies to report data on suicide and self-harm (encompassing both non-suicidal self-injury [NSSI] and/or suicide attempt).

Studies were excluded if they measured associations between alcohol use and suicidal outcomes without evaluating the effect of a specific policy [12,13,14,15]. Studies were also excluded to avoid duplication where the sample either partially or fully overlapped with that of other studies included in the review [16,17]. This exclusion criterion particularly affected studies on the effect of the anti-alcohol Perestroika campaign in individual states (countries) of the former Union of Soviet Socialist Republics (USSR) [18,19].

Studies were selected for inclusion using a two-stage process: (1) titles and abstracts were screened independently by KW and RW; (2) full texts were reviewed against the inclusion and exclusion criteria to determine eligibility by KW, RW, and KC independently. Disagreements were resolved through consensus discussions with KK.

### 2.3. Data Extraction and Synthesis

For each study, we extracted information on: (1) study information; (2) methodological characteristics, and (3) details of the intervention—alcohol policy approach. In categorizing the alcohol policies, we followed the World Health Organization’s (WHO) recommended target areas for policy action at the national level, which have been proposed in WHO’s global strategy to reduce the harmful use of alcohol [20].

Given that we anticipated few studies would report sufficient numerical data to enable meta-analysis, particularly with regards to mortality of suicide in the control (or historical) comparator condition, we elected to undertake a systematic review of results from these studies.

### 2.4. Risk of Bias Assessment

Risk of bias in the included studies was assessed using the Risk Of Bias In Non-randomized Studies of Interventions—of Exposures (ROBINS-E) tool [21].

## 3. Results

**Figure 1:**
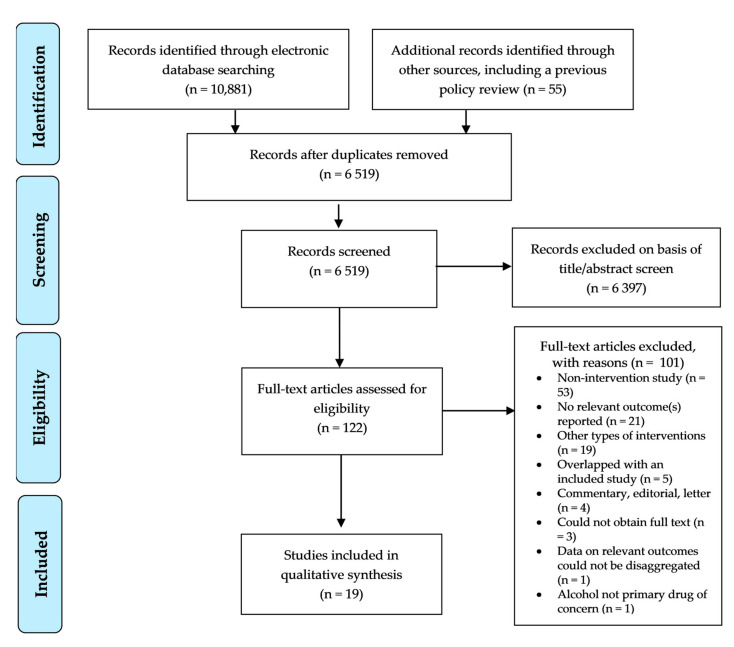
PRISMA Flowchart.

We located 10,881 records. A further 55 relevant studies were identified through ancestry-searching. Following de-duplication, 6519 individual records remained, with 6397 records being excluded after title and abstract screening. The remaining 122 full-text records were assessed for eligibility with 19 papers remaining in the final review (Figure 1).

Nineteen papers investigated the effect of changes to alcohol policies on suicidal behavior. All studies were ‘natural experiments’ analyzing the impact of changes in alcohol policies on suicide rates using mainly time-series analysis with a before and after or a quasi-experimental design on the population-level (Table 2). The majority of studies analyzed suicide mortality; only one used self-harm admissions to the general hospital as an outcome measure. Nine papers examined the effects of alcohol availability [22,23,24,25,26,27,28,29,30], seven changes to alcohol pricing [28,29,31,32,33,34,35], three changes to drink-driving countermeasures [28,29,36] and four investigated the effects associated with change in alcohol policy including multiple measures [37,38,39,40]. Two papers analyzed different measures separately and are therefore included [28,29], whilst two other papers, despite reporting on the same intervention over similar time periods, presented data for different eligible outcomes (i.e., suicide [22] and non-fatal self-harm [26]). The majority of these studies were conducted in the US [22,23,24,26,28,29,31,35,36], followed by the USSR [39,40], Canada [30], Denmark [33], Lithuania [38], Russia [32], Slovenia [37], Switzerland [34], Sweden [27], and the UK [25]. Two papers about the same policy from the USSR were included as they were separated by gender [39,40]. Different data presentation and analysis methods did not enable meta-analysis and a narrative analysis following the WHO recommended target areas for policy action at the national level [20] was conducted.

### 3.1. Alcohol Availability

There were a variety of different policy components examined that specifically addressed the impact on suicidal behavior associated with various restrictions on alcohol availability in the form of enforcing minimum legal drinking age (MLDA), dram shop laws, restrictions on hours of trading, privatization, outlets, and complete alcohol bans.

A US study examining the MLDA on suicide rates compared states with a younger MLDA of 18 years to those with an older age requirement of 20–21 years [23]. The study compared youth suicide across the 48 states (1970–1990); states with younger MLDAs had 8% higher suicide rates amongst 18- to 20-year olds and 6% higher rates in 21- to 23-year olds, even following adjustment for a number of indicators of socioeconomic disparity [23]. No significant effects were found for adolescents below the MLDA.

Findings from studies examining varying degrees of alcohol bans have been mixed. A study from the US found that implementing a ‘dry’ law (i.e., prohibiting the sale and importation of alcohol in the community) was associated with increased suicide rates [24]. However, another analysis using more complex modelling showed that a higher proportion of dry counties is associated with the lower level of suicides in males aged 20 to 24 [28]. Two more recent studies investigated the effect of differing restriction policies implemented across a number of Native Alaskan communities. Communities selected the option of adopting either: a ‘dry law’, a ‘damp law’ (i.e., less restrictive controls on the sale and/or importation of alcohol in the community) or a ‘wet law’ (i.e., no restrictions are placed on the sale and/or importation of alcohol in the community) [22]. Implementing either law was associated with a 10.3% reduction in suicide rates; however, the effect was greatest following the introduction of a ‘damp law’ as compared to a ‘dry law’ (63.0% versus 4.9%) [22]. There was no significant difference between communities in rates of hospitalized self-harm cases [26].

A study investigating the effect of liberalization of alcohol licensing laws in the form of extending trading hours for bars and public houses in Scotland found an increase in hospitalizations for self-poisoning with co-ingested alcohol in both genders [25]. Privatization of the alcohol stores in Canada saw an increase in male suicides [30]. However, an analysis of dram shop law in the US showed no effect on suicides in the age group of 25–64 years [29].

### 3.2. Alcohol Pricing

Changes to policy that have resulted in price changes have been investigated for all alcohol beverages and specific beverage types. The introduction of a 2006 law regulating the production and sale of ethyl alcohol in Russia through taxation resulted in an immediate reduction in rates of suicide in males, but not females [32]. After a dramatic increase in alcohol taxation during World War I, alcohol consumption in Denmark decreased as did the number of suicides [33]. This effect was pronounced in suicides of people with alcohol dependence.

Alcohol pricing/taxation was also found to be negatively correlated with suicides in the US [29]. Changes in the pricing of specific beverage types have also been associated with changes in suicide rates in the US. An increase in beer excise was associated with a reduction in suicides in young males aged 10 to 24 years; the effect on female suicide rates was negligible [28]. Another study reported a negative correlation with wine excise, but not with beer and spirits in the age group of 25 to 64 years. However, a study including six states found that the effect of removal of state-based retail monopolies on the sales of wine produced mixed effects [31]. Whilst four states experienced an increase in suicide rates following the removal of these monopolies (Idaho, Iowa, Maine, West Virginia), two experienced a decrease (Montana, New Hampshire) [31].

An opposite effect has been found in male suicides in Switzerland, but not for females [34]. In addition, a study on the changes to alcohol law after Sweden’s entry to the European Union in 1995 found that decreased pricing/taxation was associated with decreases in suicide rates, with a greater effect on males [27].

### 3.3. Drink-Driving Countermeasures

Three studies examined the effect of drink-driving countermeasure on suicide rates, all from the US. Two studies analyzed different blood alcohol concentration (BAC) limits for young drivers [28,36] and for all drivers in the US [28]. One study found that adoption of ‘zero tolerance’ laws was associated with a reduction in suicide rates for youth aged 15–24 years. However, the authors report that these reductions were meaningful for males between 15 and 17 (10.3%) and for males between 18–20 years (7.7%) [36]. No meaningful effects were found for females, or for older age groups. Yet, the other study noted some effect on teenage girls (negative correlation; [28]). Another study analyzing mandatory jail terms for drinking under influences (DUI) showed no impact on suicide [29].

### 3.4. Mixed Policies

The remaining studies looked at the effects of change on overall alcohol policies in different countries, which incorporated several different components, including marketing restrictions, nationwide awareness-raising activities, leadership, health services response, addressing informal and illicit production, drink driving countermeasures, as well as alcohol pricing and availability.

Two papers investigated the effect of the introduction of a strict alcohol policy in 1985, alongside social changes as a result of Perestroika, on suicide rates in males [39] and females [40] in the former USSR. Restrictions included a major propaganda campaign with anti-alcohol advertising, a decrease in alcohol production, a decrease in the number of retail outlets for the sale of alcohol, time limits on sales, punishing alcohol misuse, criminalizing the production of home-distilled alcohol, and improvements in treatment [19,39,40]. Suicide rates of both sexes were positively correlated with alcohol consumption, which declined by 31.8% for males [39] and 19.3% for females [40] after the restrictions in alcohol were introduced in 1985. Similarly, a Slovenian study found that following the introduction of the ‘Act Restricting the Use of Alcohol’ in 2003, suicide rates immediately decreased by 10% amongst men, but there was no change to rates in women [37]. The Act included several measures, such as introducing a MLDA, restrictions on alcohol advertising, and reducing trading hours.

A more recent study from Lithuania found the opposite relationship: male suicide rate increased by 14.3% between 2006 and 2009, following the implementation of multiple measures, including regulations of advertising and alcohol availability, increased taxation, and drink driving countermeasures [38].

### 3.5. Risk of Bias Assessment

Risk of bias assessment was conducted using ROBINS-E and is presented in Appendix A. The majority of studies were rated as unclear risk of bias for a number of domains due to a lack of clear reporting on exposure bias, confounding bias, baseline confounding, missing data, and selection bias. Few studies assessed and adjusted data where necessary, for temporality and seasonality, which has a major influence on suicide rates [42]. Even fewer adjusted for other influences on suicide rates, such as age, gender/sex, and socio-economic deprivation distributions. However, the assessment of risk of bias in these studies is complicated by the lack of clear guidance on evaluating bias in studies of exposures [43].

## 4. Discussion

This study systematically reviewed literature on the impact of alcohol policies on suicidal behavior and identified 19 relevant papers. The reviewed studies were ‘natural experiments’ analyzing mainly changes in alcohol policies and their effect on suicide rates using time-series analysis with a before and after design or a quasi-experimental design. Overall, the effect of societal changes in alcohol consumption through alcohol policies on suicidal behavior were studied: (1) by examining the effect of decreased access to alcohol (assumed to be associated with decreased alcohol consumption); and, (2) by examining the effect of increased access to alcohol (assumed to be associated with increased alcohol consumption). It is important to highlight the differences between these approaches because, while the assumed effect on alcohol consumption on both is clear and opposing, the underlying political purpose behind these two changes is vastly different. The intended effect of implementing more restrictive alcohol policies is to reduce alcohol-related harms in the community; conversely, relaxing alcohol laws are done for political/economic purposes.

The studies included here predominantly investigated the impact of restrictions on alcohol availability and increased cost of alcohol, and the majority of such studies found associations with reduced suicides across Western and Eastern Europe, as well as the US. Hence, while not specifically implemented as a suicide prevention strategy, the policy changes were associated with the intended effect of reducing a form of alcohol-related harm. Indeed, regulating pricing and availability of alcohol are considered as ‘best buy’ measures of an alcohol policy by the WHO, meaning they are effective, cost-effective, and feasible [20]. There were some studies that investigated changes in suicide rates associated with the introduction of an alcohol policy with multiple strategies. These studies are harder to disentangle with regards to individual strategies that may aid in reducing suicide rates. Two interventions were associated with reductions in suicide rates in the former USSR [39,40] and in Slovenia [37]. Despite analyses of the strict alcohol measures during Perestroika in the former USSR seeing over 30% decline in male suicide rates in Lithuania (as a part of the former USSR [39]), a more recent study from Lithuania found the opposite effect with increases in suicides between 2006 and 2009 after an anti-alcohol campaign [38]. However, while we need to consider the role of the hope-inspiring social and political climate at the time of Perestroika, [39,40], we cannot ignore the impact of the Global Financial Crisis (GFC) at the time of the more recent alcohol campaign in Lithuania. Rises in suicide rates were reported in many countries across the world between 2006 and 2009 [44]. Sauliune et al. [38] refer to increases in unemployment, which was not controlled for in the analysis.

The studies that examined the effect of increased alcohol availability and decreased cost did not yield as consistent a message. One Swedish study looked at a time where alcohol prices dropped after entering the EU and found a decrease in suicide rates [27]. When interpreting this study, we must consider wider societal changes associated with entering the EU that occurred alongside this increased consumption of alcohol, such as increased immigration and increased economic prosperity. Another study found mixed effects of decreases in wine production monopolies across six states in the US, with some states displaying an associated decrease in suicide rates and some an increase [31]. When assessing beverage-specific changes, alcohol cultures must be considered; in different societies, different types of alcohol are consumed in different patterns [5]. When interpreting the effects of decreased cost of wine on suicidal behavior, the specific culture surrounding wine consumption in each location must be considered. Nevertheless, liberalization of Scotland’s liquor licensing laws was associated with an increase in hospitalized self-poisoning and an increased proportion of those admitted who had co-ingested alcohol at time of poisoning [25]. Similarly, in a three-stage privatization of alcohol sales in Alberta, Canada, each stage showed an increase in suicide rates, especially for males [30].

Across all studies, the anti-suicide effects associated with restricting alcohol use were predominant in males. This is unsurprising, given males are more likely than females to drink alcohol, develop alcohol dependence [20], and have positive BAC at time of suicide death and die by suicide [19,45]. Stronger effects in males also supports a potential causal link between the ecological associations found—if the associations between alcohol restrictions and suicide rates were spurious, we would not expect to see such a prominent difference between the sexes.

Young people are particularly susceptible to alcohol-related harm [20] and accordingly, youth suicides seem particularly amenable to alcohol policy changes such as drink-driving countermeasures and increasing the MLDA. However, studies have found significant increases in hospitalizations for both alcohol-use disorders and alcohol poisoning, as well as self-harm, as young people transition across the MLDA [46,47], suggesting that policies to increase the MLDA alone are unlikely to meaningfully reduce suicidal behavior across the age spectrum.

Worldwide, the incidence of both alcohol misuse and suicidal behavior [48] is higher amongst Indigenous peoples as compared to their non-Indigenous peers. Incorporating traditional beliefs into treatment may, therefore, represent an important first step in improving adherence and, through this, the effectiveness of treatments both for alcohol and other drug use problems [49] and suicidal behavior [50] within Indigenous populations.

### Limitations and Future Directions

The studies included were ‘natural experiments’, utilizing mainly ecological level measures; therefore, they are vulnerable to the ecological fallacy. Notable differences in alcohol polices and their components limited quantitative synthesis, as numerical data on rates of suicidal behavior prior to the intervention period were frequently not reported. In addition, different types of analytical approaches were used with majority of the studies not adjusting for potential confounding factors (e.g., unemployment, income level). As a consequence, whilst our results point to the potential anti-suicide effect of policies to restrict alcohol use, particularly in males, further work is required to elucidate the mechanisms by which this effect may occur, and particularly the role that local alcohol consumption patterns may play.

Additionally, although we have categorized the intervention approaches adopted in the included studies according to the WHO’s recommended guidelines [20], a number of studies were characterized by mixed interventions. This makes it difficult to establish which particular approach may be most effective in reducing rates of suicidal behavior and ideation at either the individual or population-level. The implementation of staged alcohol restriction policies with sufficient lag between each stage to assess suicide-related outcomes would help to identify approaches likely to be of greatest value in global suicide prevention efforts. However, given that over one-in-three coronial determinations for suicide deaths remain open beyond two years [51], the lag period required to ascertain the effect of staged interventions on suicide rates in particular would need to be considerable, highlighting the potential value of so-called ‘real time’ surveillance for these outcomes [52]. It is also important to note the impact of other societal changes coinciding with the campaigns (e.g., Global Financial Crisis) and their impacts, which were not controlled for. Another aspect to consider in future studies is the complex relationship between alcohol and other drugs in the suicidal process [53] and the need to analyze policies related other substances.

Finally, our review is limited by inclusion of English language literature and the studies included have been mainly conducted in Western settings, which limits the generalizability.

## 5. Conclusions

The studies included in the review predominantly investigated the effect of restrictions on alcohol availability and increased cost of alcohol, and majority found associations with reduced suicides across Western and Eastern Europe, as well as the US.

## Figures and Tables

**Table 1:** Electronic search strategy used in different databases for the present review.

Terms	Cochrane CENTRAL (Ovid)	Cochrane DARE (Ovid)	EMBASE (Elsevier)	Medline (Ovid)	Pro Quest	Psyc INFO	Pub Med	SCOPUS	Web of Science
((alcohol adj3 consum *) OR (alcohol adj3 intox *) OR (alcohol adj3 polic *) OR (alcohol adj3 intervention) OR (alcohol adj3 control) OR (alcohol adj3 restrict *) OR (alcohol adj3 prevent *) OR (alcohol adj3 law) OR (alcohol adj3 legislat *) OR (alcohol adj3 pricing) OR (alcohol adj3 price) OR (alcohol adj3 tax) OR (alcohol adj3 taxation) OR (alcohol adj3 excise) OR (alcohol adj3 h) OR perestroika):ab,kw,ti	7036	47	75,494	49,014	247,782	26,608	897,037	139,770	114,016
((self adj2 harm *) OR (self adj2 injur *) OR (self adj2 inflict *) OR (intent * adj2 injur *) OR suicide *): ab,kw,ti	3935	57	102,019	69,718	1,273,227	66,145	190,687	148,277	152,837
1 AND 2	99	1	1365	874	2626	749	2589	3668	2401
Limit to humans	91	1	1249	872	104	748	2587	3319	1910
TOTAL									10,881

**Table 2:** Methodological characteristics and main findings of the ecological-level studies.

First Author	Country	Methods	Population	Intervention	Date of Implementation	Component(s) of Standard Alcohol Policy Targeted	Follow-up	Outcomes	Main Findings
Andreasson [27]	Sweden	Interrupted time series analysis (implementing auto regressive integrated moving average [ARIMA] modelling) of mortality data.	Swedish resident population, 1994 to 2002.	Abolition of monopolies on the wholesale, import, and export of alcohol, as well as lifting of limits on the private import of alcohol associated with Sweden’s entry into the European Union.	1 January 1995	Alcohol availability	8 years	**Suicide mortality:** national mortality registers.	An increase in alcohol consumption was estimated. Predicted alcohol-related harm was compared with real alcohol-related harm. Suicides showed a decreasing trend. Males: 22.5 per 100,000 in 1995 to 19.8 per 100,000 in 2002; Females: 9.2 per 100,000 in 1995 to 6.7 per 100,000 in 2002; data estimated from graphics presented in Figures 6 and 7.
Berman [22]	US (Alaska)	Study comparing an intervention community with a control community.	Residents of Alaska Native descent in either the intervention or control communities, 1980 to 1993.	State law (Alaska local option law) which enabled communities to choose between three alcohol availability policies: (1) ‘dry law’: sale and import of alcohol prohibited within the community; (2) ‘damp law’: sale of alcohol prohibited but import for personal use permitted, or sale permitted only at one specific store; (3) ‘wet law’: no prohibition on the sale or import of alcohol within the community.	1981	Alcohol availability	12 years	**Suicide mortality:** the Alaska Bureau of Vital Statistics.	The suicide decreased from 120.3 per 100,000 to 64.8 per 100,000 (−55.5) in communities selecting less restrictive measures—‘damp law’. There were no reductions in suicide rates in communities selecting more restrictive measures—‘dry law’.
Birckmayer [23]	US (48 states)	Time series analysis implementing Poisson maximum likelihood regression.	Resident population between 15 and 23 years of age in any one of 48 contiguous states, 1970 to 1990.	State laws raising the minimum legal drinking age (MLDA) following implementation of the 1986 National Highway Safety Act.	1986	Alcohol availability	Between 2 and 13 years	**Suicide mortality:** the mortality files of the National Centre for Health Statistics.	States with younger MLDAs had 8% higher suicide rates amongst 18–20 year old’s and 6% higher rates in 21–23 year olds, even following adjustment for a number of indicators of socioeconomic disparity. No significant effects were found for adolescents below the MLDA.
Carpenter [36]	US	Negative binomial regression, weighted by estimated resident population for each state.	Resident population between 15 and 29 years of age, 1981 to 1998.	State zero blood alcohol level (so-called ‘zero tolerance’ [ZT]) laws for drivers under the age of 21 years following implementation of the 1995 National Highway Systems Designation Act.	1995	Drink driving countermeasures	Up to 17 years	**Suicide mortality:** the Center for Disease Control Nation Center for Health Statistics.	Reductions in suicide were found for 18–20 year olds (6.3%), for males between 15 and 17 (10.3%) and for males between 18–20 years (7.7%). No meaningful effects were found for females, or for older age groups.
Joubert [24]	US (Alabama)	Descriptive statistics	Alabama resident population, 1978 to 1988.	Legal prohibition of the sale of alcohol from 1920s, which is still followed by some ‘dry counties’, not allowing similar sales as ‘wet counties’.	1920	Alcohol availability	11 years	**Suicide mortality:** the Alabama’s Vital Records.	Comparison between 41 ‘wet’ and 26 ‘dry’ countries showed higher mean suicide rate in ‘dry’ counties as compared to ‘wet’ counties (t_65_ = −2.24, *p* < 0.05).
Lester [29]	US (Idaho, Iowa, Maine, Montana, New Hampshire, and West Virginia)	No specific information provided.	Resident population of Idaho, Iowa, Maine, Montana, New Hampshire, or West Virginia.	Removal of state retain monopolies on wine sales.	1971 & 1973 *	Alcohol pricing (including taxation)	10 years	**Suicide mortality:** no specific information provided.	Four states experienced an increase in suicide rates following the removal of monopolies (Idaho: average increase of 1.50 per 100,000 persons per year; Iowa: average increase of 1.24; Maine: average increase of 1.06; West Virginia: average increase of 0.52). Two experienced a decrease (Montana: average decrease of 0.44 per 100,000 persons per year; New Hampshire: average decrease of 1.56)
Markowitz [28]	US	Negative binomial regression.	Resident population of the US between 10 to 24 years of age, 1976 to 1999.	Different state-based laws - excise tax on beer - outlet density per 1000 population per state - ‘dry’ counties - blood alcohol concentration limits for driving across states (0.10 g/100mL, 0.08 g/100mL and zero tolerance)	At various time points throughout the observation period	Different laws analyzed separately: - Alcohol pricing (including taxation) - Drink-driving countermeasures - Alcohol availability	23 years	**Suicide mortality:** National Center for Health Statistics’ Compressed Mortality File.	Increase in the excise tax on beer was associated with the reduction of suicide numbers in young males (10% increase in beer tax reduced suicides up to 5%), but not for females. Number of alcohol outlets increases the number of male suicides. Higher proportion of dry counties is associated with the lower level of suicides in males aged 20–24. Drunk driving laws had some impact on teenage female suicides (negative association).
Northridge [25]	UK (Scotland)	Time series	Resident population of Scotland, aged 12+, admitted to Milesmark Hospital following an episode of self-poisoning, 1971 to 1982.	Relaxation of liquor licensing laws enabling bars to remain open for longer hours, and for pubs to remain open on Sundays. A limited number of premises were also permitted ‘all day licenses’.	1 December 1976	Alcohol availability	6 years	**Self-harm:** admissions to a general hospital following an episode of self-poisoning.	Significant increase in hospital admissions of patients co-consuming alcohol for self-poisoning during the two years of liberalization of liquor licensing laws.
Pridemore [37]	Slovenia	Interrupted time series analysis (implementing auto regressive integrated moving average [ARIMA] modelling) of mortality data.	Resident population of Slovenia, 1997 to 2005.	Introduction of a law establishing a MLDA of 18 years for the purchase and consumption of alcohol, and tightening of liquor licensing laws governing what type of outlets could sell alcohol, the introduction of time limits on sales, and the prohibition of alcohol distribution from vending machines.	2003	Mixed - Regulation of alcohol advertising, - Alcohol availability	8 years	**Suicide mortality:** the Statistical Unit of the Institute of Public Health of the Republic of Slovenia.	The analyses of the effect of this new alcohol policy showed an immediate reduction in male suicide mortality in Slovenia (period of 1997–2006 was analyzed). There was a significant drop of 3.6 male suicides per month (approximately a 10% reduction). There was no effect on female suicides.
Pridemore [32]	Russia	Interrupted time series analysis (implementing auto regressive integrated moving average [ARIMA] modelling) of mortality data.	Resident population of Russia aged 15 years and older, 2000 to 2010.	Introduction of a law regulating the production and sale of ethyl alcohol and alcohol-containing products to control the availability of alcohol, and to require registration of alcohol production and distribution facilities.	1 January 2006	Alcohol pricing (including taxation)	4 years	**Suicide mortality:** Russian Federal State Statistics Service.	There was a drop of 9.2% in monthly male suicide numbers after the introduction of the new policy in Russia (period of 2000–2010 was analyzed), the impact was not significant for females.
Sauliene [38]	Lithuania	Time series	Resident population of Lithuania, between 15 to 64 years of age, 2006 to 2009.	Introductions of regulations on alcohol advertising, including the introduction of laws against drink driving the illegal import of alcohol, as well as time limits on sales. Excise taxes were increased by 20% for spirits and 10% for beer/wine.	1 January 2008	Mixed - Awareness - Marketing restrictions - Alcohol availability - Alcohol pricing - Drink driving countermeasures	3 years	**Suicide mortality:** Lithuanian Department of Statistics.	There was an increase in suicide rates from 64.2 per 100,000 in 2006 to 73.4 in 2009 for males aged 15–64 years, there was no change for females. Similarly the years of potential life lost (YPLL) due to alcohol related suicides increased for males.
Skog [33]	Denmark	Time series	Resident population of Denmark, 1911 to 1924.	Introduction of taxation on alcohol due to shortages caused by the blockade of Denmark during World War I.	WW I (Not further specified)	Alcohol pricing (including taxation)	13 years	**Suicide mortality:** Danish mortality register.	With reduction in alcohol consumption suicide numbers dropped by 19% in 1916–1920 compared to 1911–1915. Decrease was particularly pronounced (over 50%) in alcohol abusers (as defined by the coroner).
Sloan [29]	US (48 states)	Time series	Resident population of the US, aged 25 to 64 years, 1982 to 1988.	Different state-based laws - Pricing of alcohol - A dram shop laws - Mandatory jail terms for DUI	Different between states	Different laws analyzed separately: - Alcohol pricing - Alcohol availability - Drink-driving countermeasures	6 years	**Suicide mortality:** the National Center for Health Statistics.	Increase in alcohol price had a significant negative effect on suicide.Dram shop laws and mandatory jail terms for DUI did not have impact on suicide.
Son [35]	US	Time series - state level panel data	Resident population of the US, aged 25 to 64 years, 1995 to 2004.	Excise tax on spirits, wine, beer on state level	Different between states	Alcohol pricing (including taxation)	9 years	**Suicide mortality:** the National Center for Health Statistics and the Center for Disease Control and Prevention.	There was significant negative association between wine tax and suicide rate, but no association with beer or spirits tax.
Wasserman [39]	States of the former Union of Soviet Socialist Republics (USSR)	Time series	Resident population of males, 1984, 1986, 1988, and 1990.	Introduction of a very restrictive alcohol policy, *Perestroika*, encompassing: anti-alcohol advertising, a decrease in alcohol production, a decrease in the number of retail outlets for the sale of alcohol, time limits on sales, and laws enabling persons to be arrested for public drunkenness. Taxation also increased alcohol prices by around 80% (53% per litre for vodka). Producing home-distilled alcohol was criminalized.	1 June 1985	Mixed - Alcohol pricing - Alcohol availability - Health services response - Leadership, awareness & commitment - Addressing informal and illicit production	6 years	**Suicide mortality:** the All-Union State Statistical Committee of the USSR.	Aggregate level alcohol consumption was strongly correlated with a decline in male suicide rates in the former USSR from 1984 to 1990. A decline of suicide rates by 31.8% for males. The attributable fraction of alcohol for male suicides in the whole USSR was 50% of male suicides (calculated for the year prior to the campaign—1984).
Wasserman [40]	States of the former USSR	Time series	Resident population of females, 1984, 1986, 1988, and 1990.	Introduction of a very restrictive alcohol policy, *Perestroika*, encompassing: anti-alcohol advertising, a decrease in alcohol production, a decrease in the number of retail outlets for the sale of alcohol, time limits on sales, and laws enabling persons to be arrested for public drunkenness. Taxation also increased alcohol prices by around 80% (53% per litre for vodka). Producing home-distilled alcohol was criminalized.	1 June 1985	Mixed - Alcohol pricing - Alcohol availability - Health services response - Leadership, awareness & commitment - Addressing informal and illicit production	6 years	**Suicide mortality:** the All-Union State Statistical Committee of the USSR.	Aggregate level alcohol consumption was strongly correlated with a decline in female suicide rates in the former USSR from 1984 to 1990. A decline of 19.3% in suicide rates of females was observed. The attributable fraction of alcohol for female suicides in the whole USSR was 27%, (calculated for the year prior to the campaign—1984).
Wood [26]	US (Alaska)	Negative binomial regression	Resident population of 132 predominately Alaska Native villages, 1991 to 2000.	State law which enabled communities to choose between three alcohol availability policies: (1) ‘dry law’: sale and import of alcohol prohibited within the community; (2) ‘damp law’: sale of alcohol prohibited but import for personal use permitted, or sale permitted only at one specific store; (3) ‘wet law’: no prohibition on the sale or import of alcohol within the community.	1981	Alcohol availability	10 years	**Suicide mortality:** the Alaska Trauma Registry, supplemented by the Alaska Bureau of Vital Statistics.	Average annual age-adjusted rates per 100,000 population aged 15 and over for total self-harm injuries was 223 in ‘wet’ isolated Alaska Native villages and 245 in ‘dry’ isolated Alaska native villages (rate ratio of 0.91, 95% CI = 0.76–1.08). Self-harm fatality rates were 77 (‘wet’ isolated villages) and 76 (‘dry’ isolated villages).
Yamasaki [34]	Switzerland	Time series, accounting for autocorrelation using multiple regression based on an auto-regressive model.	Resident population of Switzerland, 1965 to 1994.	Changes in taxation on different alcohol products over time.	Changes in tax over time	Alcohol pricing (including taxation)	19 years	**Suicide mortality:** the OECD Health Data.	Alcohol tax had a significant positive correlation to male age-standardized suicide rates (coef = 0.042, *p* < 0.001), but there was no association for females.
Zalcman [30]	Canada (Alberta)	Interrupted time series analysis (implementing auto regressive integrated moving average [ARIMA] modelling) of mortality data.	Resident population of Alberta (Canada), aged 15+, 1976 to 1999.	Three stage privatization of alcohol retail: (1) the opening of privately owned wine stores; (2) the opening of privately owned cold beer stores and sale of spirits and wine in hotels in rural areas; and (3) privatization of all liquor stores.	Stage 1: 1985, Stage 2: 1989, Stage 3: 1994	Alcohol availability	5–14 years	**Suicide mortality:** the Statistics Canada.	Stages 1 & 2 in 1985 and 1989 were both followed by a n increase in suicide rates for both males and females, the stage 3 in 1994 was followed by an increase in suicide rates for males only.

* identified from Wagenaar & Holder [41].

## Appendix A

**Table A1:** Risk of bias assessment.

First Author	Confounding	Baseline Confounding	Selection Bias	Exposure Bias	Bias Due to Missing Data	Bias in the Measurement of Outcomes	Bias in the Selection of Reported Results	Other Bias
Andreasson [27]	***Comment:*** No statistical adjustment made for any relevant confounders. High risk of bias.	***Comment:*** Not reported. Unclear risk of bias.	***Comment:*** Not reported; however, data ascertained from Swedish population registries. Low risk of bias.	***Quote:*** “We use two alternative alcohol indicators: (i) alcohol sales and (ii) estimated total alcohol consumption (alcohol sales plus unregistered consumption as estimated from national surveys”. (p. 1097) ***Comment:*** Alcohol sales is from Systembolaget (a government-owned chain of liquor stores in Sweden) and unregistered consumption as estimated from national surveys (description of the methodology provided). Low risk of bias.	***Comment:*** Not reported. Unclear risk of bias.	***Comment:*** Outcomes ascertained from Swedish population registries. Low risk of bias.	***Comment:*** Not reported. Unclear risk of bias.	***Comment:*** N/A.
Berman [22]	***Comment:*** No statistical adjustment made for any relevant confounders. High risk of bias.	***Quote:*** “To analyze the potential effect of alcohol on Alaska Native injury deaths, we first divide the study population into two groups. The experimental group consists of Alaska Natives living in Alaska communities that used the state local option to restrict alcohol (went ‘dry’ of ‘damp’) at some point between 1980 and 1993. The control group consists of communities that did not exercise the local option (remained ‘wet’) throughout the period 1980-93”. (p. 313). ***Comment:*** Not reported; however, it is likely there are further differences between those communities that chose restrictive options versus those that remained ‘wet’. High risk of bias.	***Quote:*** “To analyze the potential effect of alcohol on Alaska Native injury deaths, we first divide the study population into two groups. The experimental group consists of Alaska Natives living in Alaska communities that used the state local option to restrict alcohol (went ‘dry’ of ‘damp’) at some point between 1980 and 1993. The control group consists of communities that did not exercise the local option (remained ‘wet’) throughout the period 1980-93”. (p. 313). ***Comment:*** Not reported; however, it is likely there are further differences between those communities that chose restrictive options versus those that remained ‘wet’. High risk of bias.	***Comment:*** Not reported. Unclear risk of bias.	***Quote:*** “We exclude…2 of the 99 Alaska communities that passed one or more alcohol control measures because they had fewer than five Alaska Native residents in 1990”. (p. 313). ***Comment:*** Unclear risk of bias.	***Comment:*** “The coroner’s legal determination was used to classify injury deaths as accidents, suicides, and homicides”. (p. 313). ***Comment:*** Whilst some variation in coroner’s deliberations may be expected at the individual-level, this is unlikely to have significantly biased estimates. Low risk of bias.	***Comment:*** Not reported. Unclear risk of bias.	***Comment:*** N/A.
Birckmayer [23]	***Quote:*** “To control for factors that vary within the states across time or within years across states, we included in our model 3 independent variables what have been found to be associated with suicide: the percentage of a state population completing high school, the state divorce rate, and the state unemployment rate”. (p. 1366). ***Comment:*** In addition, border population, police per capita, beer tax and liquor law arrests were controlled for. Low risk of bias.	***Comment:*** Not reported. Unclear risk of bias.	***Comment:*** Not reported. Unclear risk of bias.	***Quote:*** “We obtained state-by-state legal drinking ages by reviewing each state’s statutes for changes in the state’s MLDA”. (p. 1366). ***Comment:*** Unclear risk of bias.	***Comment:*** Not reported. Unclear risk of bias.	***Quote:*** “Data for the primary outcome of the study—the number of suicide victims classified by age and year…came from the Mortality Files of the National Center for Health Statistics. Although suicides are known to be under-reported in this data set [i.e., the Mortality Files of the National Center for Health Statistics], there is no reason to expect that the depress of underreporting of suicide is related to a state’s MLDA”. (p. 1366). ***Comment:*** Unclear risk of bias.	***Comment:*** Not reported. Unclear risk of bias.	***Comment:*** N/A.
Carpenter [36]	***Comment:*** No statistical adjustment made for any relevant confounders. High risk of bias.	***Comment:*** Not reported. Unclear risk of bias.	***Comment:*** Not reported. Unclear risk of bias.	***Comment:*** Not reported. Unclear risk of bias.	***Comment:*** Not reported. Unclear risk of bias.	***Quote:*** “The suicide data for this study come from the CDC’s National Center for Health Statistics Mortality Detail Files”. (p. 834). ***Comment:*** Low risk of bias.	***Comment:*** Not reported. Unclear risk of bias.	***Comment:*** N/A.
Joubert [24]	***Comment:*** No statistical adjustment made for any relevant confounders. High risk of bias.	***Comment:*** Not reported. Unclear risk of bias.	***Comment:*** Compared ‘wet’ versus ‘dry’ counties. It is likely there are further differences between those communities that chose restrictive options versus those that remained ‘wet’. High risk of bias.	***Quote:*** “The listings of the 67 Alabama counties that were “wet” or “dry” came from the Alabama County Data Book, 1986”. (p. 296). ***Comment:*** Unclear risk of bias.	***Comment:*** Not reported. Unclear risk of bias.	***Quote:*** “Alabama’s Vital Records (Alabama Department of Public Health, 1978 through 1988) served as the source of data for the annual suicide…rate”. (p. 296). ***Comment:*** Low risk of bias.	***Comment:*** Not reported. Unclear risk of bias.	***Comment:*** N/A.
Lester [31]	***Comment:*** No statistical adjustment made for any relevant confounders. High risk of bias.	***Comment:*** Not reported. Unclear risk of bias.	***Comment:*** Not reported. Unclear risk of bias.	***Comment:*** Not reported. Unclear risk of bias.	***Comment:*** Not reported. Unclear risk of bias.	***Comment:*** Not reported. Unclear risk of bias.	***Comment:*** Not reported. Unclear risk of bias.	***Comment:*** N/A.
Markowitz [28]	***Quote:*** “Each model includes a number of other state-level factors which may influence the number of suicides over time. These variables include the female labor force participation rate, the unemployment rate, real income per capita, the percentage of the population living in rural areas, and the percentage of the population 25 years and older that has obtained a bachelor’s degree…All models include state and time dummies…The percentage of each state’s population identifying with certain religions…is also included” (p. 39). ***Comment:*** Low risk of bias.	***Comment:*** Not reported. Unclear risk of bias.	***Comment:*** Not reported. Unclear risk of bias.	***Quote:*** “Beer taxes come from the Beer Institute’s *Brewers Almanac…* [for the] number of retail outlets…these data come from *Jabson’s Liquor Handbook…*” (p. 39). ***Comment:*** Unclear risk of bias.	***Comment:*** Not reported. Unclear risk of bias.	***Quote:*** “Data on completed suicides come from the National Center for Health Statistic’s Compressed Mortality File”. (p. 38). ***Comment:*** Low risk of bias.	***Comment:*** Not reported. Unclear risk of bias.	***Comment:*** N/A.
Northridge [25]	***Comment:*** No statistical adjustment made for any relevant confounders. High risk of bias.	***Comment:*** Not reported. Unclear risk of bias.	***Comment:*** Not reported. Unclear risk of bias.	***Comment:*** Not reported. Unclear risk of bias.	***Comment:*** Not reported. Unclear risk of bias.	***Quote:*** “All patients aged 12 and over with self poisoning are admitted to the acute medical unit”. (pp. 1466–1467). ***Comment:*** Most self-harm in the community involves self-injury, and few present to acute services following an episode. High risk of bias.	***Comment:*** Not reported. Unclear risk of bias.	***Comment:*** N/A.
Pridemore [37]	***Comment:*** Whilst ARIMA modelling was used to adjust for time trends, no statistical adjustment made for any relevant confounders. High risk of bias.	***Comment:*** Not reported. Unclear risk of bias.	***Comment:*** Not reported. Unclear risk of bias.	***Comment:*** Not reported. Unclear risk of bias.	***Comment:*** Not reported. Unclear risk of bias.	***Quote:*** “The dependent varies in this study were…suicide counts…cause of death is determined by the doctor who treated by decedent and is then confirmed by a coroner or pathologist. Deaths are classified according to the International Classification of Diseases, 10^th^ Revision (ICD-10), with suicides coded as X60 to X84”. (p. 916). ***Comment:*** Whilst some variation in coroner’s deliberations may be expected at the individual-level, this is unlikely to have significantly biased estimates. Low risk of bias.	***Comment:*** Not reported. Unclear risk of bias.	***Comment:*** N/A.
Pridemore [32]	***Comment:*** Whilst ARIMA modelling was used to adjust for time trends, and results were age and sex adjusted, no statistical adjustment made for other relevant confounders. High risk of bias.	***Comment:*** Not reported. Unclear risk of bias	***Comment:*** Not reported. Unclear risk of bias	***Comment:*** Not reported. Unclear risk of bias	***Comment:*** Not reported. Unclear risk of bias	***Quote:*** “Deaths are classified according to the International Classification of Diseases, 10thRevision,22 with suicides coded X60-X84. Data for this study were obtained via a specialtabulation of anonymous death records collected by the Russian Federal State Statistics Service”. (p. 2022). ***Comment:*** Low risk of bias.	***Comment:*** Not reported. Unclear risk of bias.	***Comment:*** N/A.
Sauliene [38]	***Comment:*** No statistical adjustment made for any relevant confounders. High risk of bias.	***Comment:*** Not reported. Unclear risk of bias.	***Quote:*** “The analyses for this study were based on data for the entire country”. (p. 459). However, “[o]nly individuals aged 15–64 years were included in this study…” (p. 459). ***Comment:*** High risk of bias.	***Comment:*** Not reported. Unclear risk of bias.	***Comment:*** Not reported. Unclear risk of bias.	***Quote:*** “The 2006–2009 computerized database of the Lithuanian Department of Statistics provided information on deaths were injuries are recorded as the underlying cause of death”. (p. 459). ***Comment:*** Unclear risk of bias.	***Comment:*** Not reported. Unclear risk of bias.	***Comment:*** N/A.
Skog [33]	***Comment:*** No statistical adjustment made for any relevant confounders. High risk of bias.	***Comment:*** Not reported. Unclear risk of bias.	***Quote:*** “Data…in Denmark during the period 1911–1924…” (p. 1190). ***Comment:*** Low risk of bias.	***Quote:*** “…victims drinking status (abuse or not) was collected by a public commission…The drinking status was determined on the basis of coroner’s report”. (p. 1190). ***Comment:*** Unclear risk of bias.	***Comment:*** Not reported. Unclear risk of bias.	***Comment:*** Not reported. Unclear risk of bias.	***Comment:*** Not reported. Unclear risk of bias.	***Comment:*** N/A.
Sloan [29]	***Comment:*** No statistical adjustment made for any relevant confounders. High risk of bias.	***Comment:*** Not reported. Unclear risk of bias.	***Comment:*** Not reported. Unclear risk of bias.	***Comment:*** Not reported. Unclear risk of bias.	***Comment:*** Not reported. Unclear risk of bias.	***Quote:*** “The mortality data were based on…cause of death information abstracted from all death certificates filed with vital statistics offices in each state. Cause of death was classified according to the 9^th^ Revision of the International Classification of Diseases (ICD-9-CM)”. (p. 456). ***Comment:*** Unclear risk of bias.	***Comment:*** Not reported. Unclear risk of bias.	***Comment:*** N/A.
Son [35]	***Quote:*** “The state dummy variables deal with time-invariant differences among the states, and year dummies deal with the effects attributable to time-series differences”. (p. 110)***Comment:*** Models were further adjusted for real and lagged per capita income, outlet density rates, unemployment rate, college rate, black rate and male rate, justifications for all adjustments were discussed. Low risk of bias.	***Comment:*** Not reported. Unclear risk of bias.	***Quote:*** “This study covers all but 18 controlled states in the United States for the years 1995 through 2004 (the lagged independent variables are included in 1994) and limits its analysis to death rates of persons between the ages of 25 and 64”. (p. 104). ***Comment:*** High risk of bias.	***Quote:*** “State excise taxes for spirits, wines, and beer were obtained from the Tax Foundation web-site (http://www.taxfoundation.org/). The tax foundation publishes state excise taxes on alcoholic beverages annually”. (p. 105) ***Comment:*** Low risk of bias.	***Comment:*** Not reported. Unclear risk of bias.	***Quote:****:* “The causes of injury deaths were categorized according to the categorization of International Classification of Disease, revision 9 (ICD-9) for the years 1995–1999 and revision 10 (ICD- 10) for the years 2000–2004. The multiple Cause-of-Death data is published and released by the National Center for the Health Statistics (NCHS) and the Center for Disease Control and Prevention (CDC) [5]. The National Bureau of Economic Research (NBER) provides death data to the public” (p. 105). ***Comment:*** Low risk of bias.	***Comment:*** Not reported. Unclear risk of bias.	***Comment:*** N/A.
Wasserman [38]	***Comment:*** No statistical adjustment made for any relevant confounders. High risk of bias.	***Comment:*** Not reported. Unclear risk of bias.	***Quote:*** “…we chose to analyze self-destructive behavior on the part of men…” (p. 306). ***Comment:*** Includes only data for males. High risk of bias.	***Comment:*** Not reported; however, likely that data on alcohol consumption were obtained from the same data source as in Wasserman et al. 1998. Low risk of bias.	***Comment:*** Not reported. Unclear risk of bias.	***Quote:*** “…statistics on violent death were based on death certificates issued by forensicdoctors. In these certificates, the cause of death was described in words, without any code being used, but the doctor was obliged to specify whether it was an accident, suicide or murder. The diagnoses on death certificates were subsequently coded by…the statistical committees of the respective republic and formed the basis for the official statistics….All data were collected by the authors from primary documents kept by the All-Union State Statistical Committee of the USSR…” (p. 307). ***Comment:*** Low risk of bias.	***Comment:*** Not reported. Unclear risk of bias.	***Comment:*** N/A.
Wasserman [40]	***Comment:*** No statistical adjustment made for any relevant confounders. High risk of bias.	***Comment:*** Not reported. Unclear risk of bias.	***Comment:*** Includes only data for females. High risk of bias.	***Quote:*** “Figures for annual consumption of pure alcohol…were taken from official sources”. (p. 27). ***Comment:*** Low risk of bias.	***Comment:*** Not reported. Unclear risk of bias.	***Quote:*** “Data on causes of death were taken from official sources…from unpublished documents kept by the All-Union State Statistical Committee…” (p. 27). ***Comment:*** Low risk of bias.	***Comment:*** Not reported. Unclear risk of bias.	***Comment:*** N/A.
Wood [26]	***Quote:*** “Given that the likelihood of serious injury occurring…is largely a function of the number of residents, it was necessary to standardize each model for the population at risk”. (p. 397). ***Comment:*** Authors also presented rates adjusted by age. However, other relevant confounders were not adjusted. High risk of bias.	***Comment:*** Not reported. Unclear risk of bias.	***Quote:*** “…all villages in what has been defined by the sampling framework of the Alaska Behavioral Risk Factor Surveillance System as the ‘Bush’ stratum…Excluded from this analysis were five communities with populations of greater than 1000 people, 11 villages that are connected to communities and to other villages by state maintained solid roads, and another 15 villages that had five or fewer residents…” (p. 395). ***Comment:*** High risk of bias.	***Quote:*** “The present study also used Landenet al’s (1997) definitionsof ‘dry’ villages as those that prohibited the saleand importation and/or possession of alcohol and ‘wet’ villages as those that allowed for the importation and/orsale of alcohol”. ***Comment:*** Unclear risk of bias.	***Comment:*** Not reported. Unclear risk of bias.	***Quote:*** “[Data were obtained] from the Alaska Trauma Registry and from the Alaska Bureau of Vital Statistics death certificate records database…” (p. 395). ***Comment:*** Low risk of bias.	***Comment:*** Not reported. Unclear risk of bias.	***Comment:*** N/A.
Yamasaki [34]	***Comment:*** Authors presented rates adjusted by age and sex. However, other relevant confounders were not adjusted. High risk of bias.	***Comment:*** Not reported. Unclear risk of bias.	***Quote:*** “This study was based on…time-series data in Switzerland…” (p. 214). ***Comment:*** Low risk of bias.	***Quote:*** “From the OECD…[t]ax on alcohol…and alcohol consumption”. (p. 214). ***Comment:*** Low risk of bias.	***Comment:*** Not reported. Unclear risk of bias.	***Quote:*** “From OECD Health Data…the standardized suicide rate for men and women…” (p. 214). ***Comment:*** Low risk of bias.	***Comment:*** Not reported. Unclear risk of bias.	***Comment:*** N/A.
Zalcman [30]	***Quote:*** “In addition to controlling for confounding effects by employing a multiple interventions design, we also used Ontario as a control area. We also used per capita consumption measures, unemployment rate and rate of AA membership per 100,000 population in Alberta as control variables to help strengthen the model design”. (p. 595–596). ***Comment:*** ARIMA modelling was further used to adjust for time trends. Low risk of bias.	***Comment:*** Not reported. Unclear risk of bias.	***Comment:*** Includes people aged 15 years and over in Alberta and Ontario as a control region. High risk of bias.	***Quote:*** “Alberta data on per capita total alcohol consumption were obtained from Statistics Canada, (Statistics Canada 1976–1999) and were expressed as litres of absolute alcohol for the population aged 15 years and older”. (p. 592). ***Comment:*** Low risk of bias.	***Quote:*** “…the data points missing between existing data points … were interpolated by cubic spline functions, a nonparametric method identified by Ferreiro (1987) for dealing with a time series that has many missing values”. (p. 594). ***Comment:*** Low risk of bias.	***Quote:*** “Age-standardized male and female suicide mortality rates…were obtained from Statistics Canada…” (p. 592). ***Comment:*** Low risk of bias.	***Comment:*** Not reported. Unclear risk of bias.	***Comment:*** N/A.
